# Low-cost adaptation options to support green growth in agriculture, water resources, and coastal zones

**DOI:** 10.1038/s41598-022-22331-9

**Published:** 2022-10-25

**Authors:** Seyni Salack, Safiétou Sanfo, Moussa Sidibe, Elidaa K. Daku, Ibrahima Camara, Mame Diarra Bousso Dieng, Koufanou Hien, Bio Mohamadou Torou, Kehinde O. Ogunjobi, Sheick Ahmed Khalil S. B. Sangare, Konan Raoul Kouame, Yao Bernard Koffi, Stefan Liersch, Moumini Savadogo, Alessandra Giannini

**Affiliations:** 1 West African Science Service Centre on Climate Change and Adapted Land-Use (WASCAL), Competence Centre, Blvd Moammar El-Khadafi, 06BP 9507 Ouagadougou 06, Ouagadougou Burkina Faso; 2 Laboratoire de Développement Agricole et Transformation de l’Agriculture (DATA), Université Thomas Sankara, Ouagadougou, Burkina Faso; 3 Urban, Disaster Risk Management, Resilience, and Land Global Practice, The World Bank, Washington, DC 20433 USA; 4 Sustainable Solutions for Africa (SSA), Blvd de la Fraternité, 08BP 81555 Agbalépédogan, Lomé Togo; 5 Laboratoire de Physique de l’Atmosphère et de l’Océan –Siméon Fongang, Ecole Supérieure Polytechnique, Université Cheikh Anta Diop, BP 5085 Dakar-Fann, Dakar, Senegal; 6 Institute of Meteorology and Climate Research, Atmospheric Environmental Research (IMK-IFU), Karlsruhe Institute of Technology (KIT), Kreuzeckbahnstr. 19, 82467 Garmisch-Partenkirchen, Germany; 7 Département Etude Et Recherches Sur l’Agriculture, l’Environnement et les Marchés (DREAM), Sahel Institute (INSAH/CILSS), BP 1530, Bamako, Mali; 8 Environment & Natural Resources Directorate, ECOWAS Commission, 101 Yakubu Gowon Crescent, Asokoro, Abuja, Nigeria; 9 Potsdam Institute for Climate Impact Research (PIK), Member of the Leibniz Association, P.O. Box 60 12 03, 14412 Potsdam, Germany; 10 Laboratoire de Météorologie Dynamique/IPSL, École Normale Supérieure, PSL Research University, Sorbonne, Université, École Polytechnique, IP Paris, CNRS, Paris, France; 11 International Research Institute for Climate and Society, The Columbia Climate School, Columbia University, New York, NY USA

**Keywords:** Climate-change adaptation, Climate-change impacts, Climate-change policy, Climate change, Climate sciences, Environmental sciences, Environmental social sciences, Hydrology

## Abstract

The regional climate as it is now and in the future will put pressure on investments in sub-Saharan Africa in water resource management, fisheries, and other crop and livestock production systems. Changes in oceanic characteristics across the Atlantic Ocean will result in remarkable vulnerability of coastal ecology, littorals, and mangroves in the middle of the twenty-first century and beyond. In line with the countries' objectives of creating a green economy that allows reduced greenhouse gas emissions, improved resource efficiency, and prevention of biodiversity loss, we identify the most pressing needs for adaptation and the best adaptation choices that are also clean and affordable. According to empirical data from the field and customized model simulation designs, the cost of these adaptation measures will likely decrease and benefit sustainable green growth in agriculture, water resource management, and coastal ecosystems, as hydroclimatic hazards such as pluviometric and thermal extremes become more common in West Africa. Most of these adaptation options are local and need to be scaled up and operationalized for sustainable development. Governmental sovereign wealth funds, investments from the private sector, and funding from global climate funds can be used to operationalize these adaptation measures. Effective legislation, knowledge transfer, and pertinent collaborations are necessary for their success.

## Introduction

Climate variability and change are becoming a critical challenge to the West African region, resulting in worsening food insecurity, natural resource degradation, and the vulnerability of socioeconomic resources inland and in low-lying coastal areas. Although natural hazards such as landslides and climate-related diseases occur in the region, hydrometeorological hazards such as floods and droughts remain the most dominant and devastating disasters^1–6^, exacerbating conflicts between farmers and pastoralists^7,8^. The projected changing climate will complicate all these patterns. Concerns about hunger, poverty, fragility and disputes over scarce resources have fuelled young people's migration to Europe and America, as demonstrated by current trends in crossing the Mediterranean Sea. Therefore, the operational implementation of adaptation actions is the most crucial pathway for enabling private and governmental actors to respond to the impacts, as well as explore and exploit climate change's transformative opportunities.

There are various adaptation options developed and applied by local communities to reduce vulnerability, but many remain on small-scale demonstration initiatives and pilot projects^9–12^. Crop adaptation options include crop diversification, the genetic development of stress-tolerant varieties, changes in cropping calendars, intercropping, the application of organic and mineral manure^12–14^, soil water conservation techniques (e.g., stone bunds, half-moon, Zai, etc.)^8^, rainwater harvesting, improved irrigation techniques, and agroforestry^15–17^. Livestock adaptation strategies have been more concerned with breeding systems that include changes in practices such as diversification, intensification, and agro-pastoralism^8^. For fisheries, there is an upsurge in the search for new aquaculture species better adapted to the impacts of sea-level rise, fish pests, and diseases^18^. Meanwhile, several soft (e.g., flood mapping) and rigid solutions (e.g., construction of groins, seawalls, etc.) are designed and implemented in coastal areas to increase resilience to climate change impacts in low-lying coastal zones areas^19,20^. Some nonstructural adaptation measures include mostly income diversification through (re) organization of communities into cooperatives/associations, use of extension services (e.g., market information systems, climate information advisory)^8,9,21^, and engagement in other less climate-sensitive activities such as informal trading, temporary migration (rural-to-urban migration).

Despite this evident but informal and sluggish progress in adaptation and widespread technological advances worldwide, several challenges hamper the large-scale implementation of adaptation actions^22,23^. The weak technological developments in sub-Saharan Africa combined with capacity-building gaps and requirements have always been barriers to the practical and successful implementation of the National Adaptation Programs of Action (https://unfccc.int/topics/adaptation-and-resilience/workstreams/national-adaptation-programmes-of-action-napa/publications-napas), the Nationally Appropriate Mitigation Actions (https://unfccc.int/topics/mitigation/resources/namas-technical-resources-and-publications) and the Nationally Determined Contributions (https://unfccc.int/process-and-meetings/the-paris-agreement/nationally-determined-contributions-ndcs/nationally-determined-contributions-ndcs).

In the specific case of West Africa, countries lack the skills to formulate bankable and result-oriented climate actions from the national plans, resulting in a regional inability to access both sovereign wealth funds and international climate finance to deliver technical services for adaptation to climate change (e.g., capacity development, policy research, and recommendations, project proposal development, etc.). With the increasing global arrangements for funding, operational implementation, monitoring, and evaluation of adaptation and mitigation actions^22,24^, we update the facts and figures concerning climate change impact to inform the inter-sectoral priority needs, adaptation options, the costs for adaptation measures, and frameworks for sustainably mainstreaming these adaptation options in the green growth pace of the agricultural sector, water resources, and coastal zones of West Africa.

## Results

### Pluviometric and thermal extremes

Historical and ongoing developments of the West African climate indicate progressive warming, changing trends, and complex weather patterns depicted by near-surface in situ measurements, satellite observations, human perceptions, and climate model simulations^3,12,25^. Subject to regional warming, the West African Monsoon (WAM) system creates hotspots of extreme weather events along the coastline, home to densely populated, low-lying cities and economic hubs along the arid/semi-arid regions^4,5,25^. As a result, there has been a decreasing trend in the number of cool nights and more frequent warm days, warm nights, and heat waves^25^. On the local scale of the Soudan/Sahel subregions, a new pattern of rainfall regime has emerged since the 1990s, characterized by a mixture of pluviometric extremes during single rainy seasons, including false onsets and early cessation of cropping seasons, heavy rain events, and long dry spells^4,5^. Along the Guinean zone, the observed stationarity of the rainfall regime can be attributed to a more intense second rainy season, associated with a late withdrawal of rains and with more significant interannual variability of rainfal^26^.

**Figure 1:**
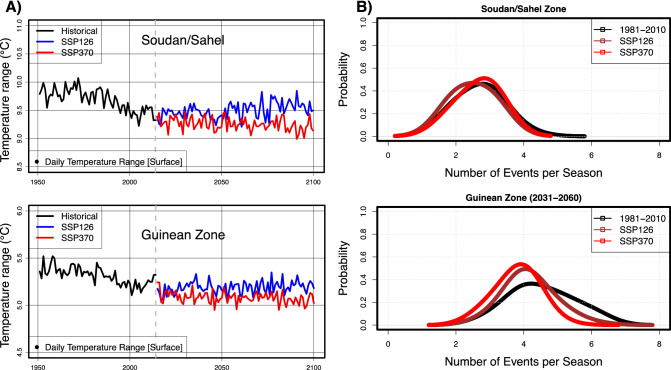
(**A**) Interannual variability and trends of the daily average temperature range (DTR) based on the near-surface temperature depicted in the Soudan/Sahel and Guinean zones of West Africa. The 1950–2014 series is based on observations combining in situ and gridded data. Projections over 2015–2100 are derived from the shared socioeconomic pathways SSP126 & SSP370 scenario using five bias-corrected and downscaled global circulation models from the intersectoral impact intercomparison project (ISIMIP3b). (**B**) Average seasonal distribution of heavy rain events (HRE) during the 2031–2060 horizon over the climatic zones of West Africa.

Analysis of state-of-the-art climate change scenarios reveals that temperature will continue increasing, the diurnal temperature range (DTR) will continue to reduce (Fig. 1A), with an increase in average air temperatures (Supplementary Fig. S2a), and an increased frequency of heat extremes such as scorching days and hot nights^25^. It is well established that high nighttime temperatures significantly impact crop production by decreasing photosynthetic function, sugar, and starch content, increasing respiration rate, suppressing floral development, and accelerating crop maturity^31^. These findings are consistent with previous assessments based on global and regionally downscaled climate model output^27,69,108^. A consensus is also observable in the region's average rainfall increase and more extreme weather conditions. The Soudan/Sahel zone will likely experience an overall increase in average rainfall within 10% to 20% or more, albeit with a decrease, with a spatial dipole configuration in its western subregions covering Senegal, The Gambia, and Guinea-Bissau.

On the contrary, the Guinean zone will likely experience a quasi-stationary rainfall regime (Supplementary Fig. S2b). This pattern, corroborated in previous assessments^28,29^, is also observable in the latest simulations of CMIP6 models^27,30^. Daily rainfall events are likely to become more intense but less frequent in combination with longer intraseasonal dry spells (Fig. 1B), causing other agroclimatic extremes and compound events like false onset and early cessation of cropping seasons, shorter cropping season, farm inundation, droughts, and heat stress for staple crops (e.g., Millet, maize, sorghum, cowpea, and tubers). In addition, floods associated with heavy rain events and large storms (stronger wind gusts) will become more common. Furthermore, SSP126 and SSP370 show that agricultural droughts are more frequent without a clear spatial pattern on the time horizons of 2031–2060 and 2071–2100 (Supplementary Fig. S3). This mixed dry/wet pattern of the rainfall regime was attributed to global warming through internal variability of the dynamic factors of the regional atmosphere and the warming of the ocean surface temperature^31^. At the same time, the dominance of greenhouse gases explains future projections of wetter conditions in the Soudan/Sahel zone^32^. Many other weather-related risks are associated with temperature extremes, although other climate variables such as rainfall, wind gusts, radiation, and humidity compound the challenges.

### Needs for adaptation in crop, livestock, and fishery production systems

According to the consensus of scenarios, without adaptation measures a wide range of changes in cereal yield is reported, with an overall average yield reduction unevenly distributed over a region for grains (e.g., pearl millet, sorghum, maize, and rice), tubers and root crops, groundnuts, cowpea, soybeans, and cotton under different rainfall regimes (Supplementary Fig. S4). From Supplementary Fig. S4, pearl millet is less sensitive to warming rates below two degrees among cereals, partly due to its solid coping capacity in adverse conditions. For rice varieties, whose growth cycle is assumed to be invariant over the actual and projected future needs, a slight increase in yield of about + 9% could be observed. However, for cases where a shortening of the growing cycle induced by higher temperatures is accounted for, decreases in yield could reach − 42% relative to reference periods^33^. Yam production is also expected to decrease by 28% for every 1 °C increase, because warming contributes to the proliferation of pests and diseases, affecting yam growth, development, and production^34,35^.

Meanwhile, cassava is less negatively impacted by climate change (− 3.7% to + 17.5%) as it is tolerant to drought and is not easily damaged by heavy rains^36^. However, floods were found to be detrimental to cassava yields. There is an inverse relationship between cassava yield and climate parameters with a significant negative effect of increasing maximum temperature. Generally, soybeans and groundnuts are less affected by rising temperatures, since photoperiod sensitivity limits the duration of crop development and the CO_2_ fertilization^37^. By the end of the century more than 60% of bean-growing areas will have to transition to alternative crops and legumes (e.g., soybean and groundnut), representing promising agribusiness opportunities under climate change^38^. Future regions suitable for growing crops are relocated to the Soudan-Savanna for groundnuts and maize, while millet and sorghum remain applicable across all agroecological zones. Cotton production benefits primarily from chemical fertilizers distributed by national cotton companies and favored by private sector investments. When integrated soil-crop management and high mineral fertilizer levels are used, future cotton productivity is expected to increase slightly, by approximately + 7 to + 31% in all future climate change scenarios^39,40^. However, large amounts of rainfall during the germination stage are unfavorable for cottonseeds, and rainfall variations during the maturity stage could significantly affect yield^40^.

Therefore, water availability and the predictability of crucial factors such as the onset, duration, and cessation of rainy seasons will be critical for cotton production in West Africa. Large parts of production areas will become unsuitable for farming in the future and will need to be converted^41^. This highlights a strong differentiation of climate vulnerability within the cocoa belt, for example, as areas in the western, central, and eastern regions will likely become hotter and wetter^41,42^. The most vulnerable areas are near the forest-savanna transition in Nigeria and eastern Côte d'Ivoire. On the contrary, the least vulnerable areas are in southern parts of Ghana, Côte d'Ivoire, and Liberia^42,43^. Interestingly, cashew and shea nut trees will likely be strained by dynamic climate change factors (e.g., increased frequency and intensity of winds, intense rainfall, increase in temperature) and unfavorable diseases^44–46^.

The change will negatively affect livestock through the impacts of heat stress on animal performance^47^, water availability, quality, pastoral resources^8,48,49^, reproductive performance, milk, and meat production^48,50^, livestock mobility (i.e., redistribution of pastures and corridors), and animal diseases^49,50^. The length of consecutive days of heat stress for dairy cattle, with intensities higher than severe and dangerous thresholds, is likely to increase (Supplementary Fig. S5). Under future climate conditions, the average length of periods with extreme and dangerous heat stress is expected to grow from ~ 3 days in the historical period to ~ 4–7 days by 2021–2050 and even to up to 10 days by 2071–2100. Around 22% of the dairy cattle population is also expected to experience approximately 70 days of more severe/dangerous heat stress, especially in the southern half of West Africa^50^. Therefore, significant decreases in productive and reproductive performance will be within − 22% relative to the 1981–2010 baseline. Thus, the interaction between transhumant herders and host communities intensifies competition over natural resources (e.g., transhumance corridors), triggering conflicts resulting from heat stress and water availability due to climate extremes.

Inland fisheries and aquaculture will also be indirectly affected by climate change through the depletion of resources in water bodies, the proliferation of parasites and ichthyotoxic plants^19,20,51^, inundation, and salinization of land and coastal freshwater due to the rise of sea level (SLR), increasing the frequency of floods, storms, and storm surge expected in riparian and coastal zones^51^.

### Needs for adaptation in hydrology and water resources management

**Figure 2:**
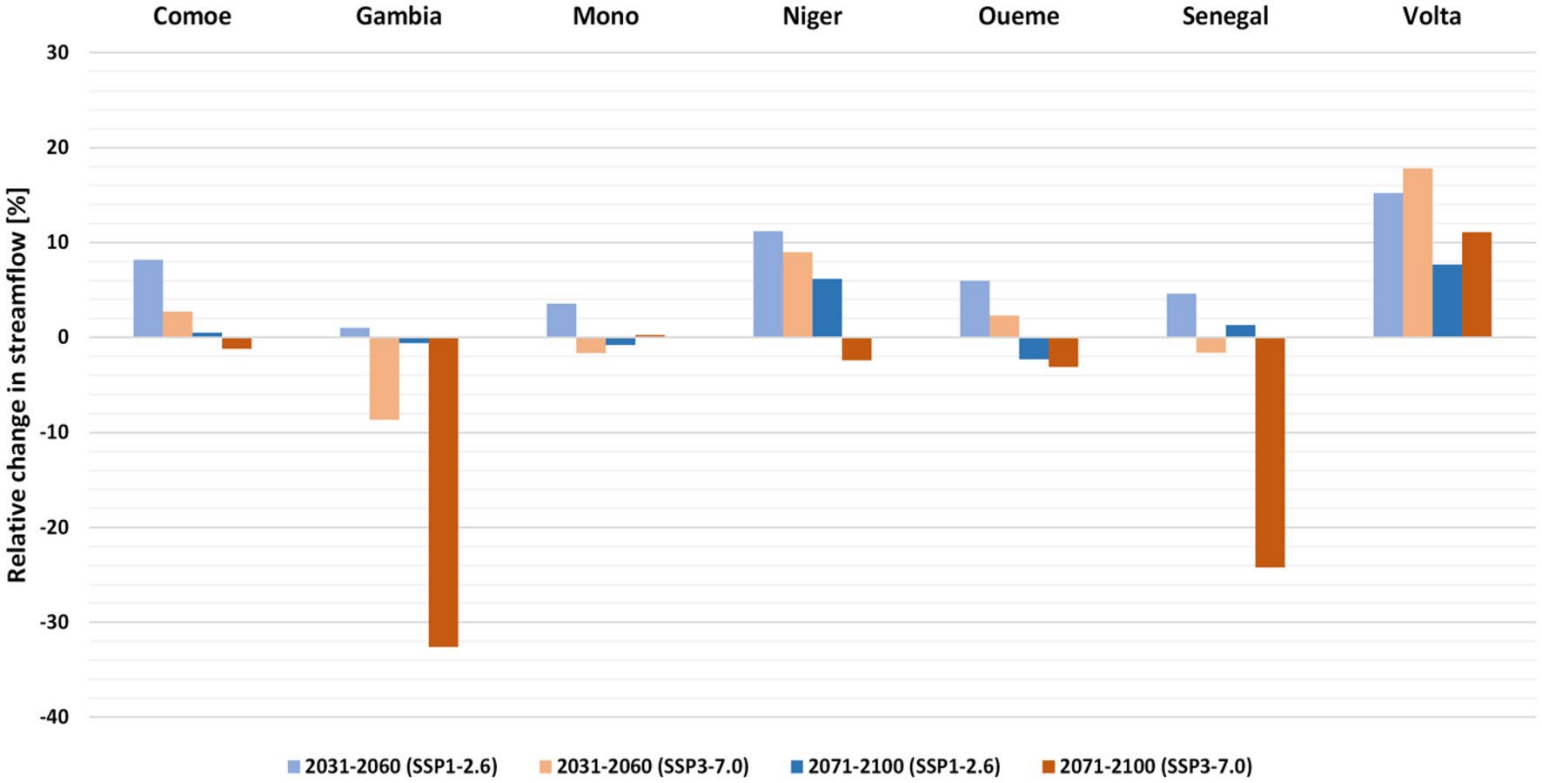
Changes in streamflow (%) relative to the 1981–2010 baseline for seven major river basins in West Africa.

Climate change is expected to impact river basins and water supply systems significantly. Floods associated with intense rainfall have become more common in the region  since the 1990s, with substantial human insecurity, including damage to production, communication, transport systems, and health and livelihoods^3,12^. Furthermore, the projected demographic pressure in West Africa (~ 800 million estimated population by 2050) will increase demand, pose substantial threats to water security^2^, and significantly modify hydrological regimes and natural ecosystems. The main rivers in the region showed minor changes in discharge on a regional scale, with median results (different time horizons, scenarios, and models considered) within the range of ± 5%^52–54^ (Supplementary Fig. S7). At the basin scale, all major rivers show an increasing trend in surface flow^52,55^, except for the Gambia and Senegal river basins, which exhibit a significant negative trend of 8–16% and 22–26%, respectively, consistent with the drying pattern projected for precipitation in this subregion (Fig. 2). The overall relative change signal in most basins is an unevenly distributed relative change signal with positive and negative values. This opposite tendency was partly attributed to the structural uncertainty of the hydrological models^54^ and the reference period used^56^. Other rivers in the region often present insignificant median relative changes in discharge, implying that the impact of climate change is small or not precise, even if it could potentially be strong in some cases (Fig. 2). However, on a local scale, the results highlight zonal contrasts in median runoff changes between the western (dry) and eastern (wet) Sahel and between the north (and more robust decreases in discharge) and the southwest (pronounced increases) regions of West Africa (Supplementary Fig. S8). Most basins are experiencing flood events with magnitudes expected to increase, with alarming extremes due to high sensitivity to climatic and land use changes, improper dam management^57,58^, and settlements in flood-prone areas^59,60^.

Therefore, water sanitation and hygiene (WASH) can be affected by many forms of water pollution, such as salinization of groundwater, intrusion of sediments, organic carbon, pathogens, and pesticides that will significantly affect populations^60–62^. Groundwater, which sometimes contributes to assuaging the threat of climate change on water availability, may highlight a decrease in quality, especially for unconfined shallow aquifers that offer little or no attenuation to contaminants from the polluted surface under flooded rivers and lagoons. Although simulated changes suggest that the average recharge of groundwater storage in the central Soudan/Sahel zone may increase by the 2050s, in the western coastal regions significant decreases are predicted^63,64^. However, there is no clear trend on the impact of climate change on the coverage of drinking water supply for domestic/municipalities because groundwater contributes, in some cases, to reduce threats. Irrigation water needs are projected to increase by 15% to 30% depending on the basins by 2050 as well^63,64^. The decrease in river discharge and the increase in evapotranspiration associated with global warming could pose a severe threat to hydropower^65–67^. The development of infrastructure in river basins often involves trading off competing objectives in an uncertain environment with many transboundary and nexus issues that need to be well integrated and managed accordingly^66,67^.

### Potential impacts on the coastal zones

West African coastal areas, coastlines, and littorals are exposed to changes in ocean parameters such as sea surface warming, sea level rise, wave height, ocean acidification, flooding, and erosion^21,68^. Estimates of ocean warming along the coasts can range between 1.5–2.5 °C and 1-3.2 °C from mid to the end of the twenty-first Century. From Sierra Leone to Guinea-Bissau, the West Coast is exposed to swell ocean waves from the north and south Atlantic that are low to moderate energy^69^. The future change in the annual significant wave heights (Hs) is more important in the Guinean coastal countries in the near term, and a substantial decrease in Hs is projected over the long term. Sea level rise (SLR) threatens West African coastal communities. It is linked to coastal hazards such as storm surges, flooding of low-lying areas, coastal erosion, and damage to coastal infrastructure and ecosystems^70,71^.

SLR is the main factor affecting coastal vulnerability, estimated to be moderate to very high in some study areas^71,72^. Changes in ocean chemistry are dominated by ocean acidification, which is oxygen depletion or deoxygenation^21,73^. However, due to significant uncertainties in potential biogeochemical effects and in the evolution of tropical ocean dynamics, there is a lack of consensus on the future volume of oxygen-poor waters^74^. Coastal marine ecosystems (for example, mangroves) and fishing are already affected. They will continue to be concerned in the future, as increases in ocean warming and weakening of the upwelling due to unfavorable wind at some locations may lead to serious rippling effects on the productivity of small pelagic fish species^75–77^. Therefore, the maximum catch potential of fish stocks is projected to decline by more than 50% by the 2050s, with the most significant reductions in countries near the equator, except for countries like Cabo Verde, Gambia, and Senegal^78^. Given the SLR rates and the future predicted shoreline vulnerability calculations (Supplementary Fig. S9), West African mangroves will face the threat of being completely inundated should sea levels rise beyond the levels they can cope with (Supplementary Note 2 and Supplementary Fig. S10).

### Costs of inaction, early, and delayed actions on adaptation

**Figure 3:**
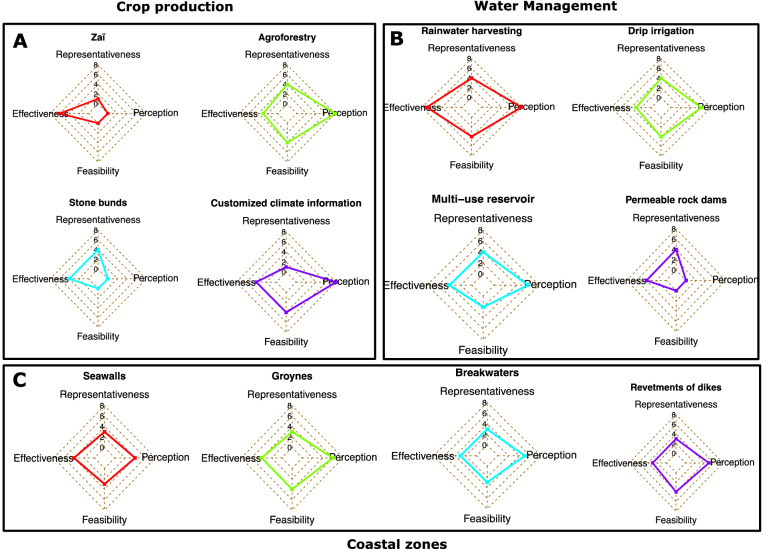
High-scoring adaptation options and measures suitable for crop production and soil water conservation (**A**), water resources management (**B**), and the protection of coastal zones (**C**) of West Africa under climate extremes.

Recent reports provide adaptation costs at global and regional levels, including an analysis of adaptation finance gaps in Sub-Saharan Africa^23^. The highest adaptation costs are projected to be needed in the water supply, coastal zone protection, infrastructure, and agriculture sectors^6,23,24^. The practices fitted to and deemed sustainable relative to the future West African climate are scored and ranked according to the practitioners' suitability, effectiveness, feasibility, representativeness, and perceptions (Tables 1 and 2). The highest-scoring adaptation options are given in Fig. 3, with much better perceptions by the local populations, ease implementation (high feasibility), and apparent effectiveness and representativeness according to a S.W.O.T-based analysis. As the impacts of climate change may also alter and shift the global cultivation area of various crops, the extent of the water body and coastlines^24^, the estimation of costs is expressed in the adequate standard unit (e.g., Hectare, Cubic meter, or Kilometer) useful for interpolations on much larger scales (e.g., countrywide, basin-scale, regional, etc.). Estimating adaptation options in agriculture, water resources, and coastal zones are classified into 'costs of inaction' and the costs associated with implementation, known as 'costs of action.' The latter consists of using the current market costs of similar options and then adjusting them to reflect market prices over time, considering depression, discount rates, interest, and projected inflation rates^79^.

**Table 1:** List of adaptation practices in crops and water resources management.

Adaptation practice	Scale*	Category**	Suitability
Stone bunds	S/M	I	Relevant for subregions with projected increase in extreme rainfall events (e.g., Central & Eastern Soudan/Sahel)
Halfmoon	S/M	T	Particularly relevant for degraded land with projected water stress (e.g., western Soudan/Sahel)
Zai	S/M	T	
Sand dune stabilization	S/M	T	The increasing speed with which desertification is progressing in Sahelian countries makes this technology one of the main instruments for combating the impacts of climate change. Suitable mainly in the semiarid subregions of West Africa
Permeable rock dams	S/M	I	Relevant for regions with a projected increase in extreme rainfall events (e.g., eastern Sahel). Serve the double purpose of prevention against erosion and provision of additional cultivated areas
Conservation tillage	S/M	T	Land conservation and restoration techniques are mostly suitable in the Soudan/Sahel regions
Grass strips	S/M	T	
Changing cropping calendar	L	M	Particularly relevant across West Africa, where models predict erratic rainfall with high interannual variability in agriculturally relevant rainfall characteristics (e.g. extreme rain events, wet/dry and hot spells)
Short-distance crop	L	M	
Crop diversification & intercropping	L	M	Ensures a more resilient planting system Particularly relevant in the context of global warming and projected changes in West Africa
Post-harvest Storage	L	T/M	Relevant across West Africa to ensure food security and adapt to erratic rainfall in the off season
Agroforestry	L	T	Tolerant to climate variability based on tolerances of perennial species
Assisted natural regeneration	S/M	T	Particularly relevant for degraded land. Mostly the Sahelian band in West Africa
mulching	S/M	T	Reduces water loss, suppresses weeds, reduces the raindrop splash effect, reduces soil temperatures, and generally improves crop productivity through the gradual addition of soil nutrients Suitable for land restoration and efficient water use. Relevant for the entire West African domain under global warming
Improved fallows	S/M	T	
Use of organic fertilizers	L	T	Particularly relevant for degraded land with poor soils. Represent a greener alternative to inorganic fertilizers
Integrated nutrient management	L	M	Requires a significant number of animals exhaust, as cattle dung is useful in making compost and micro dose of chemical fertilizers. More relevant in the marginal Sahel lands Runoff must be controlled so that compost / manure added to the soil is not lost. Combined with other practices such as stone bunds, half-moons, and permeable rock dams
Rainwater Harvesting	L	E	Relevant for the entire West African region considering projected high intraseasonal variability (e.g., heavy rains events mixed with long dry spells)
Irrigation (drip irrigation)	L	E	Water efficient irrigation techniques, suitable for predicted high water stress regions in full irrigation (off-season) and supplementary irrigation (on-season)
Multi-purpose reservoirs	L	I	Particularly relevant for transboundary and NEXUS issues. Well-fitted to improve food production, hydropower generation, irrigation needs, and water sanitation and hygiene, supply for domestic/municipalities, and the general physical and social infrastructure in rural areas
Livestock grazing space	L	T	Source of domestic animal feed, it addresses transhumance needs and agropastoral conflicts. Applicable on all regional scales and countries
Mixed crop-livestock farming	L	T/M	Provides a source of nutritional and financial security for rural populations. Relevant at the regional scale
Pest management	S/M	M	Particularly relevant across West Africa under global warming conditions, as changes in key climate variables might induce outbreak of pests and livestock diseases

*Scale: *S* small, *M* medium, *L* large.

**Category: *T* technical, *M* management, *I* infrastructure/equipment.

**Table 2:** List of adaptation practices for the coastal zones.

Adaptation practice	Scale*	Category**	Suitability
Storm surge barriers	S	I	Relevant for protection against storm surge occurrences. Might be implemented at specific locations due to the high maintenance costs
Cliff stabilization	S/M	T	Coastal management practice should be considered for application over a long stretch of coastline. Particularly relevant for countries such as Mauritania and Ghana
Flood mapping/risks assessment	L/M	T	An important process to be undertaken on the scale of West Africa to ensure preparedness. Combined solutions include raising structures above floodplains and identifying properties at risk
Floodproofing and sheltering	L	M	
Drought warning system	L	T	Particularly relevant across West Africa to provide critical information to alleviate food insecurity, water resources depletion, conflicts over natural resources, loss of life (animal and human) under climate variability and change
Construction of dikes	S/M	I	Particularly relevant for low-lying flood-prone areas. An important option to consider in West Africa, particularly in countries where extreme wave conditions are projected
Construction of seawalls	S/M	I	Relevant for countering coastal flooding or recession and erosion. Particularly relevant at the regional scale with projected increases in sea level rise
Construction of jetties	S	I	Suitable for intercepting sediments transported alongshore. Could be implemented to mitigate channel siltation
Construction of groynes	L	I	The most popular method used in West Africa to mitigate coastal erosion
Construction of breakwaters		I	Mostly implemented in ports to reduce wave actions on ships
Revetments of dikes/seawalls	S/M	T	Provide supplementary protection to existing defences such as dikes or seawall
Beach nourishment	S	T	Often adopted as a temporary solution. Very expensive option in the long term, which might be relevant only for extreme cases

*Scale: *S* small, *M* medium, *L* large.

**Category: *T* technical, *M* management, *I* infrastructure/equipment.

#### Crop production

**Figure 4:**
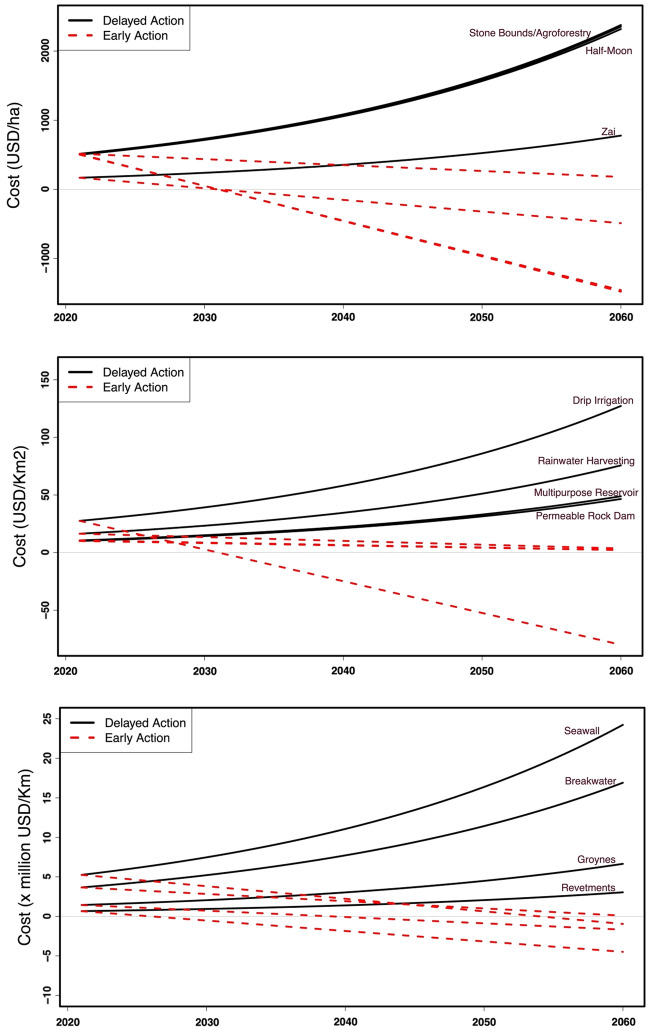
Projected costs of delayed (early with depreciation) implementation of adaptation measures when applied to crop management, water management, and the protection of coastal areas.

The impacts related to climate change on crop production vary according to crop types, climate change scenarios, and timelines. The costs of action/inaction vary depending on adaptation options. When early measures are implemented to support crop management, the project cost is expected to reduce shortly after implementation. Three categories of crops are investigated, namely cereals (for example, rice, maize, millet, sorghum), tubers and root crops (for example, Yam and cassava), and cash crops (for example, peanuts/corn). For crop production, the current implementation of agroforestry will cost USD514.9 per hectare (/ha), followed by stone bunds (USD509.77/ha), and finally, small-scale adaptation options (USD168.5/ha) (e.g., Zai techniques, Mulching, etc.), which are specific to the semi-arid zones. Going the "*Rocky Road*" (i.e., SSP370 scenario), initial inaction costs in crop production are estimated to be USD575.44/ha, and the estimate for the SSP126 method will amount to USD392.78/ha by the 2050s. Crop production losses are higher for tuber & root crops (e.g., yam) than cash crops (e.g., peanuts) and cereals. The cost of inaction in crop production is expected to soar shortly, with a larger uncertainty spectrum found in the SSP370 scenario (Supplementary Fig. S11). Due to initial investment costs, action costs appear more elevated than inaction costs. However, they will become insufficient when considering depreciation, representing 13–20% of the initial costs depending on the type of adaptation option (Fig. 4).

#### Livestock and transhumance

Transhumance is a livestock production system characterized by regular seasonal movements between complementary ecological zones under the care of a few herders. This breeding method generates conflicts between farmers in host countries and transhumant herders due to pressure on shared water and natural resources^7,48^. Several policies and laws have emerged in the different states of the West African region to reduce or prevent farmer-herder conflicts and promote sustainable transhumance. However, the operationalization of these texts is not yet a reality^47^. Projected climate extreme events will likely accentuate transhumance in the subregion where some countries such as Benin, the Ivory Coast, Togo, and Ghana have become host or transit countries^7,8,47^. Therefore, in the medium and long term, adaptation options in the livestock sector will require an innovative legal framework and the application of legislation in which multistakeholder platforms will be established for collaboration and consultations among livestock sector actors (e.g., herders, farmers, administrative authorities, local traditional rulers, land owners, researchers, etc.). Recommendations for an efficient legal framework for the sustainable management of transhumance are provided in Supplementary Table S2. For sustainable grazing spaces and corridors, goods and services may need only USD18,693.37 to build a 250-ha reference-protected reserve. However, the correct implementation of the legal framework, the monitoring, evaluation, and management system must be carried out to support rural development, reduce conflicts and improve the practice of transhumance and raising livestock (Supplementary Table S2).

#### Water resources and coastal zones

The losses and damages in the water resources sector are estimated at USD7.7 per cubic meter. Implementing multiuse reservoirs will cost only USD1.6 per cubic meter. The adoption of rainwater harvesting will cost USD2852.8 per hectare of crops, while drip irrigation and permeable rock dams will cost USD2756.0 and USD300.7, respectively. In coastal areas of West Africa, floods often result in considerable material loss and damages (e.g., damage to crops, destruction of houses, bridges, etc.). To prevent injuries or reduce effects, investing in seawalls, breakwaters, revetments, and groins will cost USD 5,250,000.0/km, USD 3,663,003.0/km USD 1,440,000/km, and USD 650,000/km, respectively. Investment costs re reduced (i.e., USD262,500, USD91,575, USD36,000, and USD26,400 per km, respectively) when considering depreciation, which corresponds to only 8–11% per year of the cost of inaction. The estimated cost of inaction will be USD 3,164,020 per km (Supplementary Table S1 and Fig. 4).

With increasingly recurring and projected hydrometeorological hazards, the growing user needs for weather, climate, and environmental services, the capacity standards of the national agencies responsible for early warning, civil protection, and disaster management must be improved to make countries weather-ready nations^6,80,81^. In 1–5 years, the investment needed was estimated at 3.4–6.9 million US dollars relative to the situation in 2019/2020 to cover permanent staff training and capacity building, small accessories to improve the working environment (e.g., internet connection, complimentary electricity supply to cover intermittent power, replace or repair computers/hardware), and data and information management networks in West African countries. The *Hydromet Initiative*^6^ (https://www.worldbank.org/en/programs/africa_hydromet_program) elaborated further investment costs to modernize national and regional organizations to gradually improve and sustain service quality. The investment needs were estimated at USD 324.5 million (including USD290 million for the member states and USD34.5 million in support of regional institutions), considering available cost estimates from existing projects in West Africa.

## Summary and ways forward

Under the increasing warming of the regional climate, the number of climatic hazards will continue to grow, including pluviometric extremes such as false onset and early cessation of cropping seasons and longer dry spells mixed with heavy rain events^31^. It is expected that when floods occur due to more intense and frequent heavy rain events^4,5^, they are likely to mix with agricultural droughts appearing without an apparent spatial coherence. These mixed wet-dry patterns of inland rainy seasons and heat waves will negatively impact agriculture and water resources. Meanwhile, the projected oceanic parameters of the Atlantic Ocean (e.g., sea surface warming, ocean acidification, sea level rise, and increased wave heights) will likely result in the very high vulnerability of coastal areas. Impediments to climate change in coastal regions may include relaxation of upwelling (i.e., reduction of phytoplankton), the decline in fishing, storm surges, beach erosions, groundwater, and inland salinization.

The operationalization and scaling up of adaptation actions remedy the expected, widespread, adverse effects of climate change into transformative opportunities for the region. Regional landscape analysis shows some evident but informal progress in local communities' adaptation practices to increase resilience to ongoing environmental and future climate-related challenges. Several well-fitted adaptation options are identified as technical, management, and infrastructure/equipment and classified for their suitability and potential implementation scales in agriculture, the water sector, and coastal zones (Table 1). Most of these adaptation options are developed in isolated actions and applied by communities to locally combat climate variability and improve their livelihoods on a smaller scale as demonstration or pilot initiatives. Technical and management practices can be globally adopted without external intervention or assistance. They cost little to operate and show strong positive effects on crop production, land, and labor productivity despite consecutive heavy rain events and prolonged dry spells, as revealed in farm case studies and pilot projects^8,12,23,82,83^. Infrastructural/equipment measures will likely require significant capital investment, as is the case in coastal areas where large-scale construction will be needed to overcome SLR, storm surges, floods, erosion, and the salinization of inland cropping land and shallow aquifers. Investment costs (action without delay) for infrastructural/equipment measures become lower considering value depreciation and the rate of loss and damages they are meant to avoid. As climate extremes increase, delayed action will strain investment and increase financial, human, and material costs. Loss and damage in agricultural, water resources, and coastal zones  will likely increase. These results are based on field evidence to guide investment and specific needs for the adaptation of West African countries against current and future climate change that is potentially full of hydrometeorological hazards while keeping a green growth pace for all.

There is a growing need to upscale most of these local climate actions to promote a sustainable green economy that reduces greenhouse gas emissions, enhances resource efficiency, prevents biodiversity loss, and promotes ecosystem services. However, before many of these adaptation practices can be implemented, short-term measures involving policy development, knowledge transfer, and relevant partnerships and frameworks must be established. Concerning associations, various actors are already engaged at all scales. These actors, including the private sector, must complement each other, avoid duplications, and foster synergies among climate actions for adaptation. The basis for building more fitted adaptation frameworks in West Africa is co-production, provision of customized, robust, and reliable information, capacity building, and communication strategy adapted to local users. The weak legal framework and legislation of the region and the political instability (including the ongoing security crisis in the Sahelian subregion of Mali, Niger, Burkina Faso, and northern Nigeria) create barriers to meeting adaptation needs and cause failures to comply with significant-scale climate actions promoting successful implementation of adaptation options^9^. Therefore, policy developments in the region should prioritize integrated water (natural) resource management focusing on the nexus approach^84^ and create legal frameworks and legislation for multi-stakeholder consultation and collaboration platforms between sectoral stakeholders (e.g., herders, farmers, landowners, decision-makers, etc.) to reduce conflicts and alleviate poverty in rural areas. As adaptation options are based primarily on local knowledge, it is well suited to apply soft measures such as gender equity^85,86^, climate services^82,83,87^, protection insurance schemes^87,88^, and nature-based solutions embedded with indigenous knowledge^89,90^, all supported by research and innovation in adaptation science^91,92^. These non-structural adaptation options can optimize the suitability, effectiveness, and potential scale integration in operational implementation over the region.

However, the significant challenges for operational adaptation include (i) producing more to feed a rapidly growing population, (ii) alleviating the adverse effects of weather and climatic extremes, and (iii) reducing the overall contribution to greenhouse gas emissions. However, all the priority adaptation options identified here are clean and aligned with the West African countries' green growth policies and economic emergence goals. Therefore, to close the gap in operationalization and upscaling, significant investment efforts should be developed at national and local levels, with funding coming from sovereign wealth funds of countries and the private sector and complemented by concerted responses at the global level.

## Data and methods

### In-situ and gridded observations

Our customized simulation designs were based on observed station data (in situ), including daily rainfall, maximum and minimum temperature, and other variables, provided by the meteorological services and agencies of the West African countries to WASCAL (www.wascal.org) following country-specific data sharing policies^93^. This assessment used in situ data from 132 synoptic and 44 discharge stations to calibrate our crop and hydrological models^94,95^ and 14 erosion hotspots in coastal zones to compute the coastal vulnerability index (Supplementary Fig. S1). The synoptic station data were quality controlled^5,34^, and the 44 daily discharge gauges data were gap-filled^94^. For climate trends and variability analysis, West Africa is divided into three subregions: the Soudan/Sahel zone, the Guinean, and the coastal zones. The West African Soudan/Sahel zone is characterized by a long dry season followed by a unique rainy season that peaks in July–August-September. The spatial distribution of total annual rainfall decreases northward from ∼1200 mm to 200 mm and is mainly concentrated over a short period of ~ 4 months. Most rain-fed cereals and a few tubers are grown in this zone. The natural factors affecting the intraseasonal variability of the rainfall regime in the Sahel include the local forcing of the Saharan dry air masses, dust, and pollution aerosols^31,96^. The *Guinean* zone is the area of a moist evergreen forest where annual rainfall is between 1500 and 1800 mm and is divided into two seasons that alternate with two dry seasons. Natural vegetation is generally grasslands and transitional woody forests. The crops are mostly maize, yam, rice, millet, sorghum, groundnuts, and cotton; sugar cane is grown in the wetter parts. Few livestock are reared, primarily trypanotolerant cattle, sheep, and goats. This subzone includes southeast Guinea, northern Liberia, Cote d’Ivoire, middle Ghana, the middle belt of Nigeria, and southern Cameroon (Supplementary Fig. S1).

The coastal zones include all coastlines, littoral, and shores of the region stretching from the Senegal-Mauritanian upwelling areas down to the southern parts of the Gulf of Guinea. The weather and climate characteristics are mainly embedded within the West African Monsoon (WAM). The regional-scale circulation features of WAM include the latitudinal movement of the intertropical convergence zone (ITCZ), the Saharan heat low (SHL), the variability of lower-to-upper-tropospheric circulation features such as the African Easterly Jet (AEJ), the Tropical Easterly Jet (TEJ), African easterly waves, and other low-level westerly jets. The global oceans also play a significant role in modulating the observed seasonal rainfall and the recent changes in the past climate signal^31,32^. The greenhouse gases are the main factors explaining the future climate patterns of the region^32^.

Other observations include gridded mean monthly precipitation (P) and minimum and maximum temperature (Tmin and Tmax, respectively) datasets for the historical period (1981–2010). They were extracted from the WATCH Forcing Data methodology applied to the ERA-Interim Data (WFDEI)^97^ meteorological forcing dataset at a grid resolution of 0.5° × 0.5°. The WFDEI data set merged with the *in-situ* data from synoptic stations to update the baseline status's temperature maps and times series (Supplementary Fig. S2a,b).

### Downscaled and bias-corrected climate change scenarios

We combined various Representative Concentration Pathways (RCPs) scenarios and the Shared Socioeconomic Pathways (i.e., SSP1 based on RCP2.6 named SSP126 and SSP3 based on RCP7.0 named SSP370)^98^. The Global Circulation Models (GCMs) used were downscaled and bias-adjusted in the Intersectoral Impact Model Intercomparison Project (ISIMIP3b, https://www.isimip.org/). The bias adjustment method is described in Lange^99^. The bias adjustment in ISIMIP3b used the WATCH-ERA5 (W5E5) data set^100^. The selection of these models was motivated by: (i) structural independence in terms of their ocean and atmosphere model components and (ii) process representation reported in an informal survey among experts as fair (IPSL-CM6A-LR, MPI-ESM1-2-HR) and sound (GFDL-ESM4, MRI-ESM2-0, UKESM1-0-LL). Moreover, the selected GCMs represent the whole CMIP6 ensemble, including three models with low climate sensitivity (GFDL-ESM4, MPI-ESM1-2-HR, MRI-ESM2-0) and two models with high climate sensitivity (IPSL-CM6A-LR, UKESM1-0-LL). Other datasets are based on Weather Research and Forecasting (WRF) and the Consortium for *Small-scale MOdelling in CLimate Mode* (CCLM) regional climate models (RCMs). These RCMs were used to downscale the GCMs under RCP 4.5 for the West African region^101,102^. Bias correction was also performed on the outputs using a multivariate bias correction (MBC), considering the interdependency between climate variables in the historical and future periods^103^. The MBC data sets were generated at a horizontal resolution of 0.11 × 0.11 degrees.

Future projections focus on two different combination scenarios representing different shared socioeconomic pathways (SSPs) and Representative Concentration Pathways (RCPs). The SSP1-RCP2.6 (SSP126), known as *'the sustainability*' scenario (i.e., Taking the Green Road), describes a world marked by strong international cooperation, prioritizing sustainable development. The underlying assumption of the radiative forcing is based on RCP2.6. It will likely keep the global temperature below 2 °C with a substantial change in land cover (increased international forest cover), low emissions, and 445 ppm carbon dioxide (CO_2_) by 2100. RCP2.6 requires CO_2_ emissions to decline by 2020 and zero by 2100. The SSP3-RCP7.0 (SSP370), known as 'the *Regional Rivalry*” scenario (i.e., A Rocky Road), depicts a fragmented world affected by competition between countries, slow economic growth, policies oriented towards security and industrial production, and little concern for the environment. The underlying assumption of radiative forcing is based on RCP7.0 with a substantial change in land use (decreased global forest cover), medium–high emissions, and 871 ppm of CO_2_ in 2100. More combinations of SSP/RCP scenarios that provide narratives describing alternative socioeconomic developments can be found in Meinshausen et al.^98^. While SSP126 represents the low end of the range of plausible future pathways, SSP370 represents the medium-to-high end of future emissions and warming^98^. For climate change signals and impacts assessments, the 1981–2010 period was considered the reference '*baseline*', and two horizons were defined for the future, namely 2031–2060 (Horizon 1) and 2071–2100 (Horizon 2). Where observed data time series is not a constraint, the assessments are conducted seamlessly over 1979–2014 (to cover the most recent historical period of observations) and 2015–2100 for future projections under SSP126 and SSP370.

### Impact assessments

#### Crop model simulation design

CERES and CROPGRO, embedded in the Decision Support System for Agrotechnology Transfer version 4.7 (DSSAT4.7, www.dssat.net)^104^, and interfaced with R software (DSSAT-R)^105^, were used to perform spatial yield simulations for millet, maize, rice and cotton productions in a changing climate. The climate forcing data files (WTH.LST) were extracted from historical (In situ observations & model outputs) and projected SSP126 & SSP370 datasets. The soil input data was extracted and formatted from ISRIC (Global Soil Information Based on Automated Mapping^106,107^, and HC27 (Global high-resolution soil profile database for crop modeling applications) generic soil profiles, soil hydraulic properties derived from pedotransfer functions^107^, and soil physical and chemical properties required by the crop models.

The cropping areas considered for each crop were digitized and are portrayed in Supplementary Fig. S5. Some regional crop management practices were also based on rainfed field regimes and applied at all points of the climate model. Millet, maize, and cotton were planted at a depth of 5 to 7 cm in a 100 × 100 cm row spacing with 2 to 3 plants m^−2^ observed at emergence. The planting dates were identified as 'the first day between April 1 and July 31 when at least 40% soil moisture is reached in the top 20 cm depth. The minimum temperature does not drop below 11 °C for millet cultivars. The maximum temperature does not exceed 35 °C’.

Organic matter estimated at 500 kg ha^−1^ of crop residues is added. Different improved hybrid rice cultivars (i.e., IR8 and TOX 3107) adapted to the rainfed conditions of West Africa were planted, with 50–100 plants.m^-2^ as the planting density observed at emergence. The planting date was the first day between May 1 and June 30 when at least 50% soil moisture is reached in the depth of the upper 20 cm, the minimum temperature does not drop below 20 °C for rice cultivars, and the maximum temperature does not exceed 35 °C. Mineral fertilizers were applied in NPK 15–15–15 at seedling at 100 kg ha^−1^ and Urea at 50 kg ha^−1^ 20 and 40 days after sowing. The output of all the simulations was analyzed considering the relative percent difference between the average yield and biomass production of rainfed millet and rice. The changes for 2031–2060 and 2071–2100 were estimated relative to the baseline from 1981–2010.

#### Hydrological modeling and coastal vulnerability simulation designs

Two hydrological models, GR4J and IHACRES^108,109^, are used to investigate the impacts of climate change on streamflow in seven main transboundary river basins (i.e., The Comoe, Gambia, Mono, Niger, Oueme, Senegal, and Volta basins) that cover more than 90% of the West African region (Fig. 3 and Supplementary Fig. S6). Both are lumped conceptual models, computationally attractive (due to few calibration parameters), and convenient for data-scarce environments. To assess the performance of these two models, the baseline period is subdivided into two subperiods representing overall dry conditions (1981–1995) and relatively wet conditions (1996–2010). To ensure the robustness of the simulations, the models are calibrated and validated during both wet and dry periods (Supplementary Note 1). The ability of models to represent hydrological regimes is assessed through the Kling-Gupta efficiency (KGE) criterion^110^.

The potential coastal vulnerability was treated by calculating the coastal vulnerability index (*CVI*)^111^. The basic physical-geological parameters include sea level rise, geomorphology, coastal slope, regional elevation, shoreline change, significant wave height, and tidal range taken from ISMI2b and ISIMIP3b datasets. Wave energy is related to erosion capacity, where relief and vertical land movements are considered flood risk indicators.

The CVI is described as the square root of the product of these physical-geological units divided by the number of variables (n).

1 $$CVI=\sqrt{\frac{a*b*c*d*e*f}{n}}$$

***a***: Geomorphology; ***b***: shoreline change rates; ***c***: Coastal slope/relief; ***d***: Relative Sea-level rate; ***e***: Significant wave height (Hs); ***f***: Tidal range. Factors *a* and *b* are obtained from the literature, *c*, *d*, and *f* are derived from satellite data, while *e* is obtained from outputs computed by the WAVEWATCH III model (https://github.com/noaa-emc/ww3) forced by the wind from coupled global models. Three of the six variables will change to calculate the CVI in the future. These variables are Hs, the sea level rise rate, and the shoreline erosion/acceleration rate. The geomorphology is obtained from literature and verified with Google Earth (https://earth.google.com/web/). Landsat images (https://www.usgs.gov/landsat-missions/landsat-data-access#C2L1) were used to calculate shoreline erosion/accretion. Sea level rise and tide data were obtained from AVISO altimetry (https://www.aviso.altimetry.fr). All these images were processed using ArcGIS 10.2 software (https://www.esri.com/about/newsroom/arcwatch/the-best-of-arcgis-10-2/) and the Digital Shoreline Analysis System (https://www.usgs.gov/centers/whcmsc/science/digital-shoreline-analysis-system-dsas). Wind speed data for wave height (Hs) calculations were extracted from the coupled climate models involved in the ISMIP3b project (https://www.isimip.org/).

The obtained CVI values are divided into four classes, with percentile ranges of 0–25%, 25–50%, 50–75%, and 75–100%. The upper quartile is taken as 'very *high vulnerability,'* the lower quartile corresponds to *'low vulnerability,'* and the remaining two classes represent in ascending order '*moderate*' and *'high vulnerability*.' Additional results of these investigations are exposed in Supplementary Note 2 and Supplementary Fig. S10.

### Analyses of costs of action and inaction

The data processed in this document emanated from various coproduction forums and surveys (e.g., series of workshops, focus group discussions and expert interviews, high-level meetings with stakeholders, and the administration of individual questionnaires to agropastoralists, farmers, households, and other users of climate services) designed to develop adaptation tools for West Africa. These were mainly conducted between January 2018 and April 2020. The data are complemented by an in-depth literature review of peer-reviewed papers and country reports^112^. Much gray literature was used in the form of consultancy reports, Ph.D. theses, conference proceedings, symposium/workshop reports, project reports, book chapters, and publicly available web sources.

Estimating the costs of adaptation involves several ramifications and complexities. In this assessment, we based our reasoning on the assumption that climate change is a "*global good*" with long-term influence, uncertainty, volatility, and some level of ambiguity. The costs of the adaptation options correspond to the investment costs of implementation and other external costs (where applicable), and the benefits are the damages, return on investment, and social benefits that the strategy is expected to avoid (i.e., crops and income losses, co-benefits from the implementation of adaptation options). An analogy method has quantified the costs associated with these damages, known as the 'costs *of inaction,'* and the expenses related to implementing the identified adaptation options, known as the 'costs *of the action.'* A cost–benefit analysis was conducted in two stages. The adaptation options are clustered based on a numerical SWOT analysis according to their representativeness, effectiveness, feasibility, and household perceptions. The S.W.O.T (*Strength-Weakness-Opportunity-Threats*) analysis was built upon a 5-point rating scale (i.e., a scale of 0, 1, 2, 3, 4) with 0 being "very poor" and four being "very good" for ‘*Strengths*’ and ‘*Opportunities*.’ For '*Weaknesses*' and '*Threats*,' the scale is negative and  inverted (i.e., − 4, − 3, − 2, − 1, 0), with -4 being high levels of threats and weakness and 0 being 'no threats’ and ‘no weaknesses.' In each case, the SWOT scores are cumulated to identify the level of representativeness, effectiveness, feasibility, and perception, with eight being the highest and 0 being the lowest. Therefore, the most highly rated adaptation options, with much better perceptions by local populations, are easy to implement (high feasibility) and have apparent effectiveness and representativeness for agriculture and water resources, and coastal zones (Fig. 3 and Tables 1 and 2).

After completing the SWOT-based clustering, these topmost sustainable adaptation options are valued from the angles of costs of action, inaction, and investments. In this paper, the focus is more on evaluating the monetary cost. The costs of the adaptation options correspond to the investment costs of implementation and other external costs (where applicable). The prices considered are based on the average regional market prices available during the 2010–2019 period for similar options in the ECOWAS countries. The costs are adjusted to reflect the projected price trends for 2021–2060, considering depreciation, discount rates, and projected inflation rates. The benefits are the damages, return on investment, and social benefits that adaptation options are expected to avoid or their suitability to alleviate the adverse effects of climate change.

Estimates cover the costs for each identified adaptation option, including large equipment, small equipment, professional labor, and non-professional labor. When the costs of an alternative have been estimated in the past, the present value of these costs is considered. Therefore, we capitalized on these values using the inflation rate in the ECOWAS zone provided by the International Monetary Fund (IMF) (https://www.imf.org/external/datamapper/PCPIPCH@WEO/OEMDC/ADVEC/WEOWORLD).

Hence, if ${C}_{n}$ is the cost of an adaptation option, to establish the capitalization value ${C}_{t}$, we add up all the flows generated by the investment as:

2 $${C}_{t }= {C}_{n} \times {\left(1+i\right)}^{t-n}$$

where ${C}_{n}$ is the value of the flow in year n, ${C}_{t}$ is the value of the flow in the previous year (year t), $i$ is the annual interest rate for risk-free investments, $t-n$ is the years between the flow payment (year n) and the previous year.

As the impacts of climate change can also alter and shift the global cultivation area of various crops^24^, the extent of the water body and the coastlines, the estimation of adaption costs are at this moment expressed in the smallest relevant standard unit (e.g., Hectare, Cubic Meter, or Kilometer). The estimated expenses cover small equipment, large equipment, and professional and non-professional labor.

## Supplementary Information

Supplementary Information.

## Data Availability

ISIMIP3b data are available for download at https://www.isimip.org/. Bias-corrected WASCAL high-resolution climate simulation datasets can be downloaded free from the WASCAL Data Infrastructure (WADI) at https://wascal-dataportal.org/2.0/. The raw, uncorrected WASCAL high-resolution simulation outputs can be downloaded at https://cera-www.dkrz.de/. Crop, hydrological, coastal vulnerability index simulation outputs, and economic estimation data are available upon request through the corresponding. Inflation rates for the 15 countries of the Economic Commission of West African States (ECOWAS) are available at https://www.imf.org/external/datamapper/PCPIPCH@WEO/OEMDC/ADVEC/WEOWORLD.

## Materials availability

The in-situ climate datasets, customized DSSAT-R scripts for the agroclimatic simulations, and the R scripts used to determine the agro-hydro-climatic parameters are available upon request to S. Salack (salack.s@wascal.org). The hydrological lumped models are open access. Parametric versions for the major river basins of West and Central Africa are also available upon request from M. Sidibé (moussa.sidibe01@gmail.com) and S. Salack. The results of the coastal vulnerability index are available upon request to I. Camara. Excel spreadsheets of cost-benefit simulations are available upon request from S. Sanfo (sanfo.s@wascal.org) and S. Salack.
