# Supplementary material for: Low-cost adaptation options to support green growth in agriculture, water resources, and coastal zones
Source: Sci Rep. 2022 Oct 25;12:17898. doi: 10.1038/s41598-022-22331-9 (PMC9596419; doi:10.1038/s41598-022-22331-9)
Supplement: Supplementary file 1 — Supplementary Information. [file 41598_2022_22331_MOESM1_ESM.docx]

**Low-cost Adaptation Options to Support Green Growth in Agriculture, Water Resources, and Coastal Zones**

***Supplementary Materials***

**Authors:**

Seyni SALACK^1,*^, Safiétou SANFO^1,2^, Moussa SIDIBE^3^, Elidaa K. DAKU^4^, Ibrahima CAMARA^5^, Mame Diarra Bousso DIENG^6^, Koufanou HIEN^1^, Bio Mohamadou TOROU^1^, Prof Kehinde O. OGUNJOBI^1^, Sheick Ahmed Khalil S.B. SANGARE^7^, Konan Raoul KOUAME^8^, Yao Bernard KOFFI^8^, Stefan Liersch^9^, Moumini SAVADOGO^1^, Alessandra GIANNINI^10,11^

**Affiliations:**

^1^West African Science Service Centre on Climate Change and Adapted Land-use (WASCAL), Competence Centre, Blvd Moammar El-Khadafi, 06BP 9507, Ouagadougou 06, Ouagadougou, Burkina Faso.

^2^Laboratoire de Développement Agricole et Transformation de l’Agriculture (DATA), Université Thomas Sankara, Ouagadougou, Burkina Faso

^3^Urban, Disaster Risk Management, Resilience, and Land Global Practice, The World Bank, Washington, DC 20433, USA.

^4^Sustainable Solutions for Africa (SSA), Blvd de la Fraternité, 08BP 81555, Agbalépédogan, Lomé, Togo.

^5^Laboratoire de Physique de l’Atmosphère et de l’Océan –Siméon Fongang, Ecole Supérieure Polytechnique, Université Cheikh Anta Diop, BP 5085 Dakar-Fann, Dakar, Senegal.

^6^Institute of Meteorology and Climate Research, Atmospheric Environmental Research (IMK-IFU), Karlsruhe Institute of Technology (KIT), Kreuzeckbahnstr. 19, D-82467 Garmisch-Partenkirchen, Germany.

^7^Département Etude et Recherches sur l’Agriculture, l’Environnement et les Marchés (DREAM), Sahel Institute (INSAH/CILSS), BP 1530, Bamako, Mali.

^8^Environment & Natural Resources Directorate, ECOWAS Commission, 101 Yakubu Gowon Crescent, Asokoro, Abuja, Nigeria.

^9^Potsdam Institute for Climate Impact Research (PIK), Member of the Leibniz Association, P.O. Box 60 12 03, D-14412 Potsdam, Germany.

^10^Laboratoire de Météorologie Dynamique/IPSL, École Normale Supérieure, PSL Research University, Sorbonne Université, École Polytechnique, IP Paris, CNRS, Paris, France.

^11^International Research Institute for Climate and Society, The Columbia Climate School, Columbia University, New York, NY, USA.

***Corresponding author:**

Email addresses: [salack.s@wascal.org](mailto:salack.s@wascal.org) / [abutawakalt@gmail.com](mailto:abutawakalt@gmail.com) (S. Salack).

**Supplementary Figures**

**
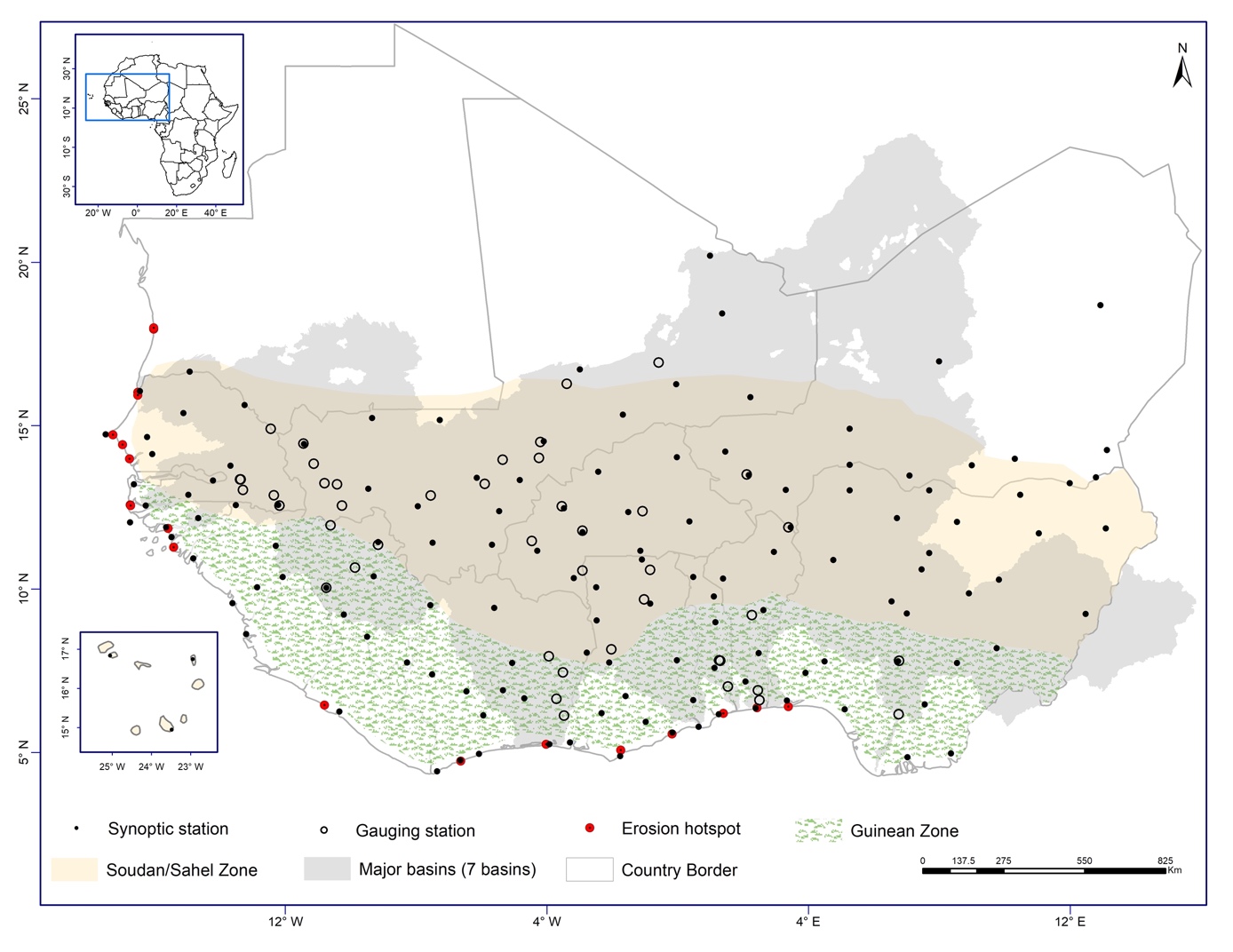
**

**Supplementary Figure S1 |** Eco-hydrological delineation of West Africa showing the limits of the Soudan/Sahel zone and the Guinean area. *In situ* data are taken from 132 synoptic stations (black dots) and 44 discharge stations (open circles), and 14 erosion hotspots in coastal areas (red dots). The gray-shaded area represents the total surface covered by the seven major river basins: Comoe, Gambia, Mono, Niger, Ouémé, Senegal, and Volta.

**Supplementary Figure S2a |** Maximum, Minimum, and Mean Air Temperature Anomalies. **Panel A:** Spatial distribution of minimum and maximum temperature anomalies relative to the 1981-2010 baseline. The shaded areas are statistically significant anomalies at a 95% confidence interval based on a *Mann-Kend test*. **Panel B.** Interannual variability and trends of the average air temperature (2m) area over the Soudan / Sahel and Guinean zones in West Africa. The shaded area represents the 95 % confidence interval across models.

**Supplementary Figure S2b | Rainfall anomalies. A) Spatial pattern of rainfall anomalies in West Africa.** The shaded areas are statistically significant anomalies at a 5% threshold using a *Mann-Kend test*. **Panel B. Interannual variability and trends of rainfall anomalies standardized relative to the 1981-2010 baseline.** The shaded area represents the 95 % confidence interval across models.

**Supplementary Figure S3 |** Frequency of agricultural droughts relative to the baseline period 1981-2010 of SSP126 (**A**) and SSP370 (**B**).

**Supplementary Figure S4 |** Rates of changes in grain yields (%) relative to the 1981-2010 baseline period as consensus impacts of different climate change scenarios on food crops in irrigated and rainfed regimes in West Africa.

**Supplementary Figure S5 |** Future category of severe and dangerous heat stress under shared socioeconomic pathways, SSP126 and SSP370, for 2031-2060 and 2071-2100.

**Supplementary Figure S6 |** The current cropping areas digitized from OCDE/SWAC [1] to implement the crop simulation design.

**
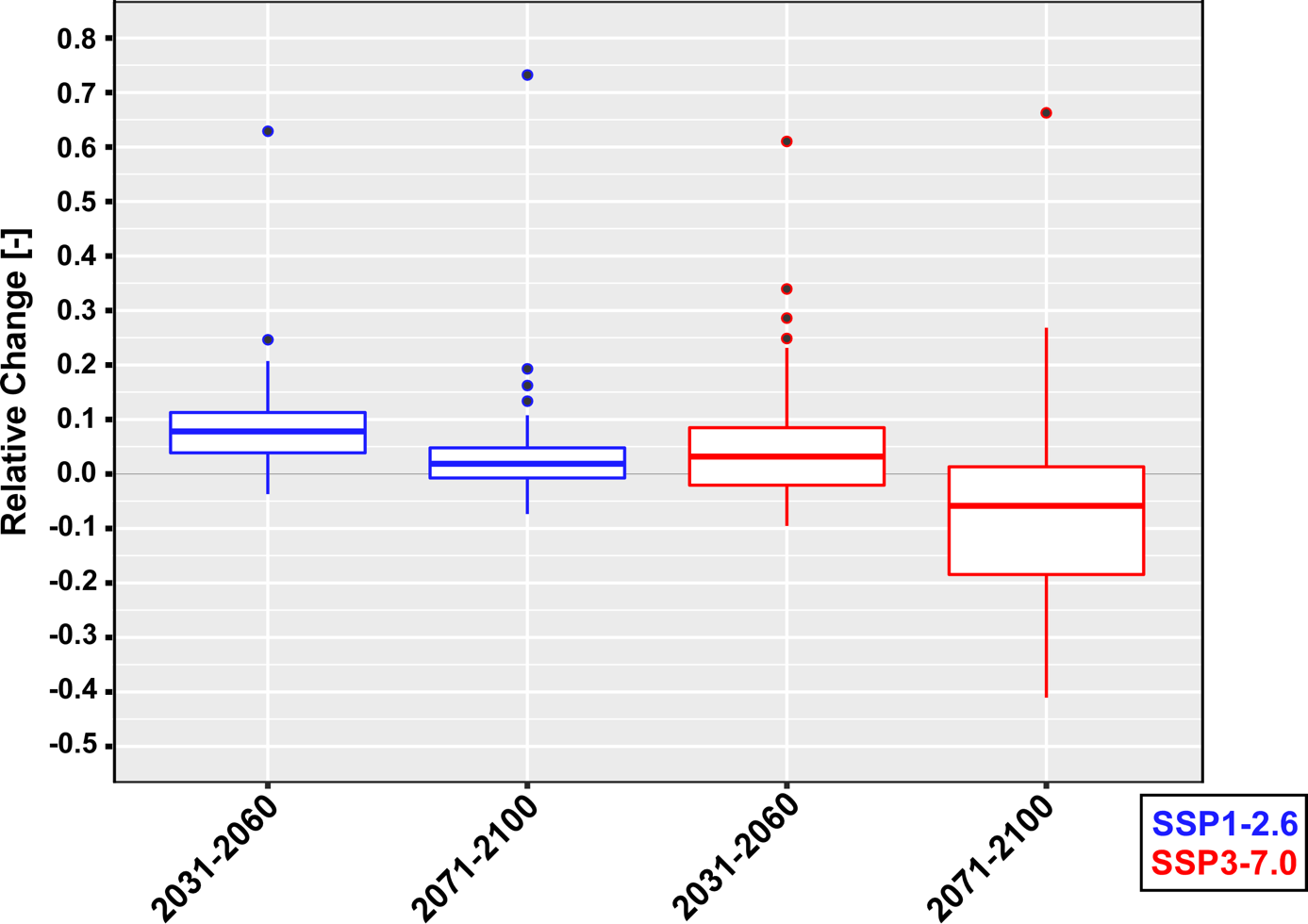
**

**Supplementary Figure S7 |** Relative change in streamflow simulated by two hydrological models (i.e., GR4J and IHACRES) averaged over the West African domain for 2031-2060 and 2071-2100 under SSP126 and SSP370.


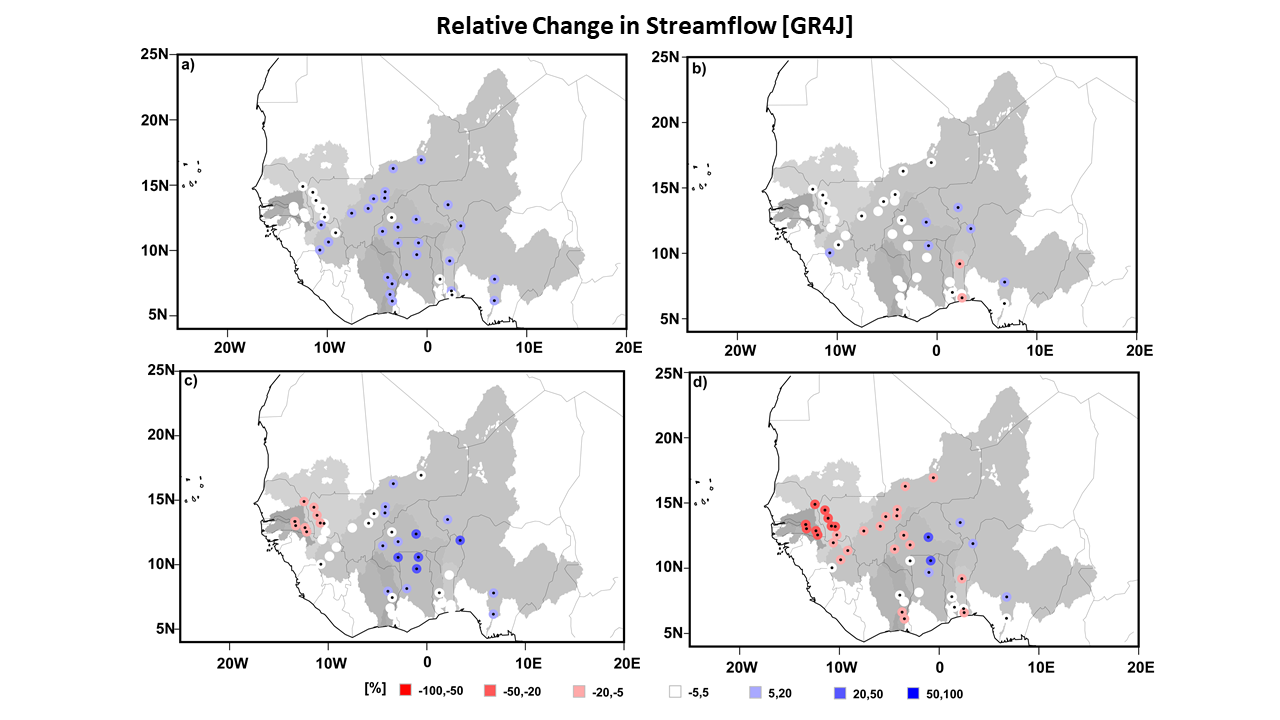


**Supplementary Figure S8 |** Relative change in streamflow for seven major river basins in West Africa using the GR4J model (a) SSP1-2.6 for the period 2031-2060, (b) SSP1-2.6 for the period 2071-2100, (c) SSP3-7.0 for the period 2031-2060, and (d) SSP3-7.0 for the period 2071-2100.


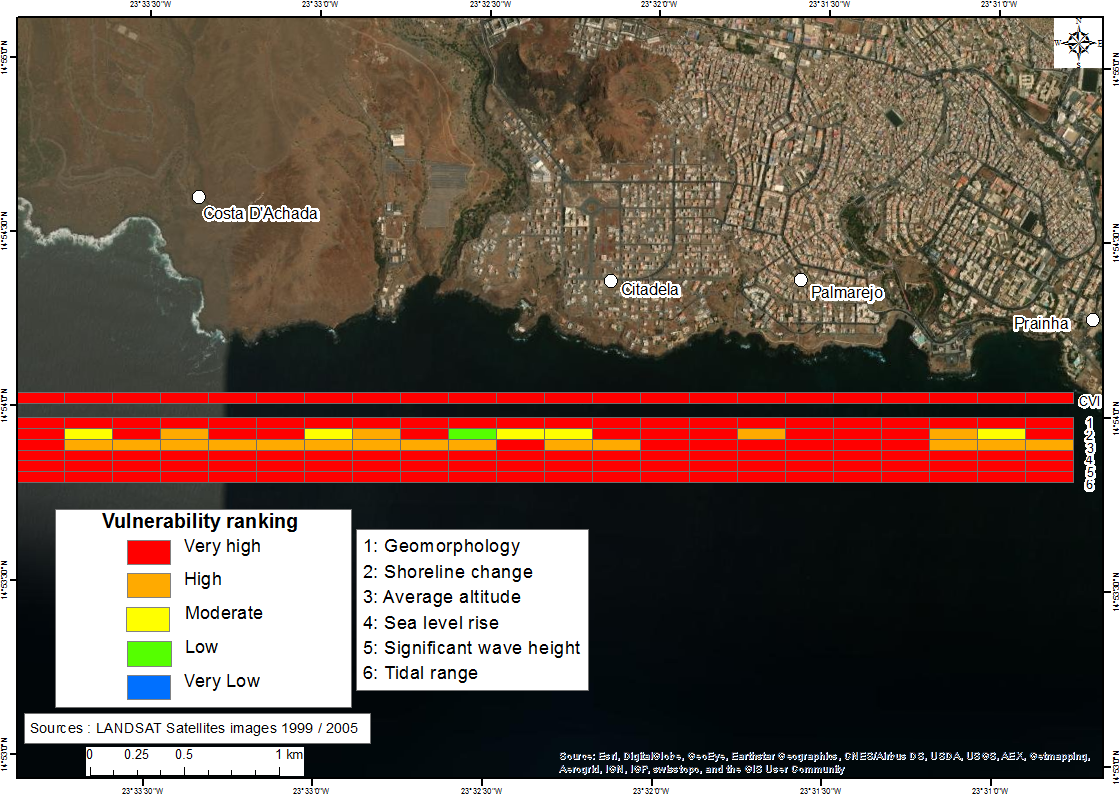

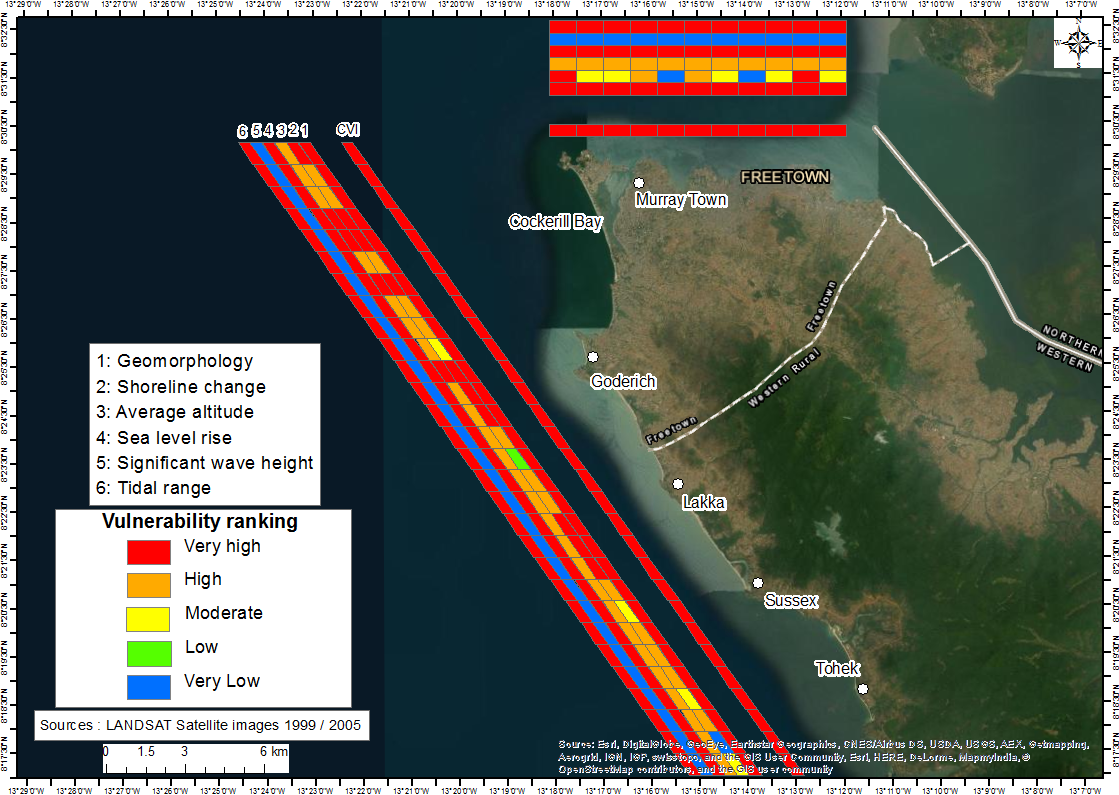


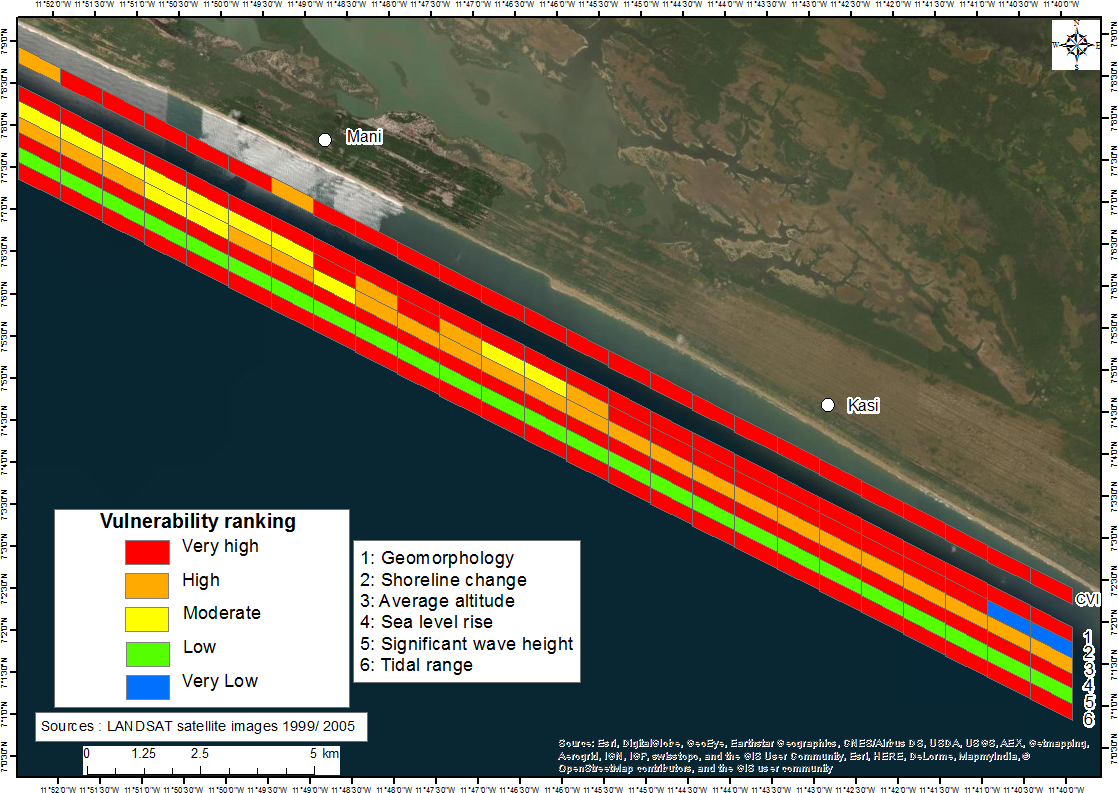

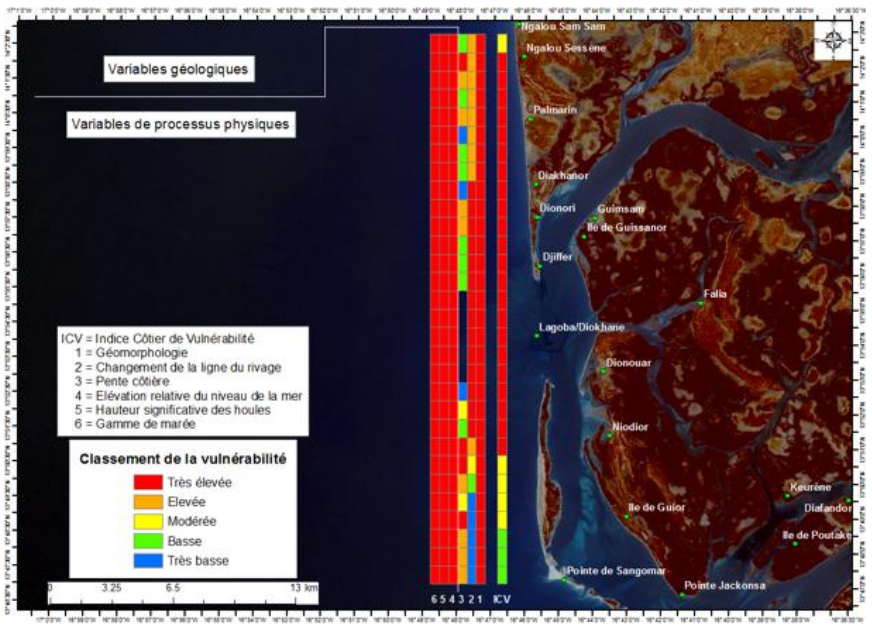


**Supplementary Figure S9|** Coastal Vulnerability Index (CVI) for the near future period (2031-2060) in Cap-Verde (upper left panel), Freetown (upper right), Lake Mape (lower left), and South Senegal (lower right). The CVI is calculated as the square root of the product of six ranked variables. These variables are geomorphology, shoreline erosion/accretion rate, coastal slope, relative sea-level rise rate, mean wave height, and mean tide range. The geomorphology is obtained from literature and verified with google earth (https://earth.google.com/web/). Landsat images (<https://www.usgs.gov/landsat-missions/landsat-data-access#C2L1>) were used to calculate shoreline erosion/accretion. Sea level rise and the tide were obtained from AVISO altimetry data (<https://www.aviso.altimetry.fr>). All these images were processed using ArcGIS 10.2 software (https://www.esri.com/about/newsroom/arcwatch/the-best-of-arcgis-10-2/) and Digital Shoreline Analysis System (<https://www.usgs.gov/centers/whcmsc/science/digital-shoreline-analysis-system-dsas>). Wind speed data for the wave heights (Hs) calculations were extracted from the coupled climate models involved in the ISMIP3b project (https://www.isimip.org/).

**Supplementary Note 1| Description of IHACRES and GR4J models**

In this study, two hydrological models (GR4J and IHACRES) are used to investigate the impacts of climate change on streamflow in seven main river basins in West Africa. Both models are computationally attractive (due to few calibration parameters) and convenient for data-scarce environments.

**The IHACRES** model is a lumped conceptual model that simulates the rainfall-runoff responses of watersheds to total streamflow with parameters calibrated before simulation through comparison with observed streamflow data^2,3^. The conceptual layout of this model shown in Fig. 4 is built upon a nonlinear loss module that converts rainfall, P, into adequate rain, U, and a linear unit hydrograph module, consisting of two parallel stores which generate streamflow. The nonlinear loss module uses a catchment moisture deficit (CMD) accounting scheme that partitions rainfall into drainage (adequate rain), evapotranspiration, and changes in catchment moisture^4^. The linear unit hydrograph (UH) module links good rainfall to streamflow with the parameters ***τq***, the quick flow response (in days); s, the regular decay time constant (in days); ***τs***, the slow flow response (in days); and ***νs***, the proportional volumetric contribution of slow streamflow. The configuration of stores is identified from the time series of rainfall and discharge. Still, this configuration typically consists of only one store representing ephemeral streams or two parallel stores representing base or slow and quick flow. This approach reduces parameter uncertainty inherent to hydrological models while simultaneously attempting to characterize internal hydrological processes^3^. This model implemented for the Niger River basin by Oyerinde et al. [5] produced satisfactory results.

**The GR4J** model is a rainfall-runoff model that is based on four free parameters: X1, the maximum capacity of the production store (mm); X2, the groundwater exchange coefficient (mm); X3, the maximum capacity of the routing store (mm); and X4, the time peak ordinate of the hydrograph unit UH1 (day). The production store (X1) is stored at the soil surface that holds rainfall. The storage capacity depends on the types of soil in the river basin. Low porosity in the soil can increase the size of the production store. The groundwater exchange coefficient (X2) is a function of groundwater exchange, which influences the routing store. Negative values of X2 indicate water infiltration into the aquifer, while positive values suggest that water exits the aquifer and adds to storage in storage in the routing. The routing storage (X3) is the amount of water stored in soil porosity. The value of X3 depends on the type and humidity of the soil. The time peak (X4) is when the ordinate height of the flood hydrograph is created during the GR4J modeling. The ordinate of this hydrograph is generated based on runoff, where 90% of the flow is a slow flow that infiltrates the ground, and 10% is a fast flow along the soil surface. The schematic diagram of this model based on Perrin et al. [6] is presented in the figure below

The baseline period is subdivided into two periods representing overall dry conditions (1981-1995) and relatively wet conditions (1996-2010). To ensure the robustness of the simulations, the models are calibrated and validated in both wet and dry periods. The ability of models to represent hydrological regimes is assessed through the Kling Gupta Efficiency (KGE) criterion (Equation below).

$$KGE=1-\sqrt{\left( r-1 \right)^{2}+\left( \alpha-1 \right)^{2}+\left( \beta-1 \right)^{2}}$$

Where r is the linear correlation between observations and simulations, α is a measure of the error of flow variability, and β is a bias term. Taking the mean flow as a benchmark, performances in the range -0.41 ≤ KGE ≤ 1 could be regarded as ‘reasonable’ because the model outperforms this benchmark^7^

**Supplementary Note 2 | Marine Ecosystems, Fishing, and Coastal Vulnerability**

The West African coastline is very dynamic, with rocky coasts representing only 3 percent of the beach. The remainder of the coastline is mainly composed of mangroves and sandy formations, which offer little resistance to the action of coastal currents^7^. The coastal zones of West Africa are very low-lying, vulnerable, and expected to be the worst hit by the impact of SLR induced by climate change. Many coastal areas are experiencing accelerated degradation related to erosion and flooding due to extreme climatic factors^9,10,11^. From 1968 to 1986, coastal areas were characterized by accretion in Senegal. The opposite was observed between 1986 and 2004 when a more significant recession was recorded. The recession remained a decisive element of the coast from 2004 to 2017, though on a slower retreat scale^9^. In Sierra Leone, mainly in Freetown, more erosion hotspots are found on the southern coast than on the northern coast. The maximum erosion rate oscillates between 10 to 18 m / year, whereas the complete accretion on the south coast is around 33 to 10 m/year. In Guinea Bissau, our results reveal erosion along the coast. The maximum (minimum) erosion rate is 252 m/year (78m/year). In Cap-Verde, the change in shoreline rate is less significant. The maximum rate of erosion is around nine m/year. In Mauritania, more precisely in Nouakchott, coastal erosion could lead to a loss of -7.66 km2 in 2050 under CP 4.5 and -27.3 km2 under RCP 8.5. In the same scenario, a loss of -40.6 km2 could occur by 2100^12^.

In Senegal, by 2080, three-quarters of the Senegalese coastline will be exposed to a high risk of erosion, compared to the current figure of 25%. The risk of flooding due to sea storms, which is already very high (more than 50% of the coastline is at high risk), is expected to cover two-thirds of the coast by 2080. At the mouth of the river in Senegal, the city of Saint-Louis represents the most extreme example of the combination of risks. This city is already subject to a significant risk of flooding, which would intensify with rising sea levels, preventing the flow of river water into the ocean. By 2080, 80% of the city could be submerged every year. On the southern coast of Senegal, in Saly Portugal, a loss of land of 225.56 m^2^ (232.90 m^2^) is projected under the medium (large) emission scenarios by 2050^11^. By 2050, the entire linear of the coastal fringe of the Fatick region (Senegal) will be subject to the phenomenon of erosion, and likely, some coastal bangs could even disappear. This retreat will be 268.08 m, with an annual erosion rate of 8.65 m/year. The San Pedro coastline in Côte d’Ivoire could lose an area of 0,23 km^2^ (0,39 km^2^) in 2050 (2100) due to coastal erosions. These losses exacerbate the Abidjan coastline. The combined impact of SLR and the highest high tide by 2050 and 2100 was considered the worst permanent flooding possible in Mauritania, Senegal, Côte d’Ivoire, Togo, and Benin.

Coastal vulnerability is investigated under the combined effect of six physical impact climatic drivers in coastal zones using the coastal vulnerability indicator (CVI) method. Supplementary Figure 10 below illustrates the CVI for the period 1981-2005 in some coastal cities of West Africa. On the coast of Cap Verde, CVI values ranged between 0.32 and 2.84 from 1981 to 2005. These values are classified into four vulnerability classes (Figure in the upper left panel). The results indicate a very high vulnerability front in the cities of Prainta, Citadela, and Costa D’achada. An increased vulnerability is found on the Palmarejo coast. These increased exposures are mainly due to ocean parameters, wave height, and tidal range. However, just west of Citadela, we showed low vulnerability due to a moderate rate of shoreline change and an increase in sea level. The coastal exposure of the coastline along Lake Mape and Freetown in Siera-Leona is also investigated (Figure below). These choices were motivated by a National Park and essential settlements. For Lake Mape, a high to very high vulnerability is found on both sides of Kasi. West of Mani city, the vulnerabilities range between low and moderate. This is mainly due to the change in the shoreline rate and Hs. In Freetown coats, the southern part is more vulnerable than the northern part. In the latter area, the vulnerability is moderate to low, while we found higher to very high pixels on the southern coast. This difference is mainly due to the rate of change in the shoreline, which presents more erosion on the south coast due to exposure to waves and storms. A cape protects the northern coast.


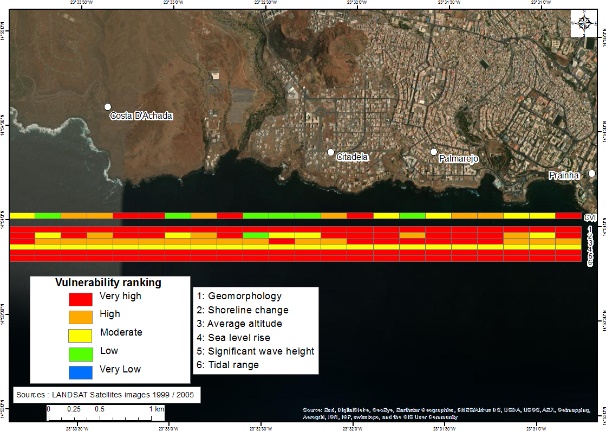

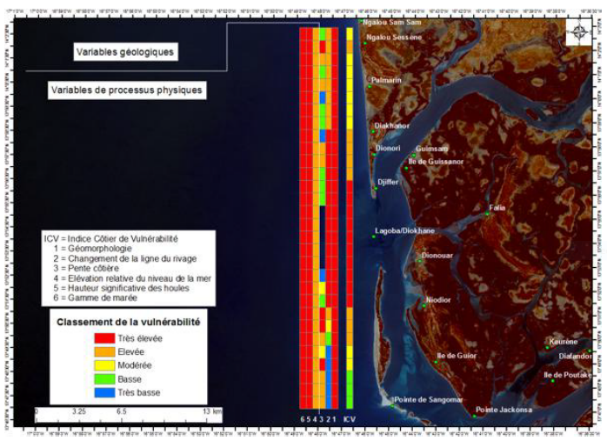

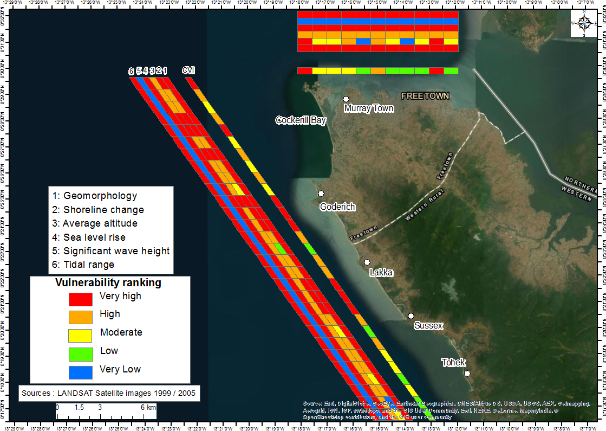

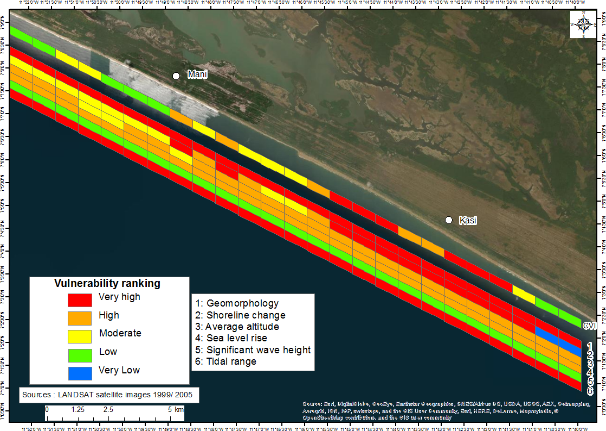


**Supplementary Figure 10 |** Coastal vulnerability index (CVI) for the actual period (1981-2005) in South Senegal (upper left), Cap-Verde (upper right), Freetown (bottom left), and Lake Mape (bottom right). The CVI is calculated as the square root of the product of six ranked variables. These variables are geomorphology, shoreline erosion/accretion rate, coastal slope, relative sea-level rise rate, mean wave height, and mean tide range. The geomorphology is obtained from literature and verified with google earth (https://earth.google.com/web/). Landsat images (https://www.usgs.gov/landsat-missions/landsat-data-access#C2L1) were used to calculate shoreline erosion/accretion. Sea level rise and the tide were obtained from AVISO altimetry data (<https://www.aviso.altimetry.fr>). All the images were processed using ArcGIS 10.2 software (https://www.esri.com/about/newsroom/arcwatch/the-best-of-arcgis-10-2/) and Digital Shoreline Analysis System (https://www.usgs.gov/centers/whcmsc/science/digital-shoreline-analysis-system-dsas). Wind speed data for wave height (Hs) calculations were extracted from the coupled climate models involved in the ISMIP3b project (<https://www.isimip.org/>).

**Supplementary Figure S11** | Cost of inaction on cereals (e.g., Millet, sorghum, maize, rice), groundnuts, cowpea, yam, and cassava in West Africa.

**Supplementary Tables**

**Supplementary Table S1 |** Costs of inaction and action on adaptation options in ECOWAS coastal zones

**Supplementary Table S2 |** Costs of goods and services for the sustainable development of pasture space and transhumance corridors for livestock

**Supplementary Table S1 | Average initial** costs of Action & Inaction on adaptation options in the ECOWAS countries of West Africa.

|  | **Inaction**  **(SSP126)** | **Inaction**  **(SSP 370)** | | | **Cost of action** | | | |  |
| --- | --- | --- | --- | --- | --- | --- | --- | --- | --- |
| **Crops Production (USD/ha)** | | | | | | | | |  |
|  | |  | |  | Customized Climate Services | | Agroforestery | Stones Bunds | Zai |
| Cereals | | 13.8 | | 16.4 | 247.64 | | 514.90 | 509.77 | 168.50 |
| Tubbers | | 1,122.2 | | 1,371.6 |  |  |  |  |  |
| Cowpea / peanut | | 28.6 | | 338.3 |  |  |  |  |  |
| **Water Resources Management** | | | | | | | | | |
|  | |  | |  | Multi-use reservoir (USD/m^3^) | | Rainwater Harvesting (USD / m3) | Drip irrigation  (USD/ha) | Permeable Rock Dams (USD/ha) |
| Flooding, Irrigation needs | | - | | - | 1.64 | | 10.08 | 2,756.00 | 300.70 |
| **Costal Zones (USD/km)** | | | | | | | | | |
| Flooding, Erosion, Salinization, Sea level rise, Ocean waves | | 3,164,020 | | 3,164,020 | Breakwaters | | Seawalls | Groynes | Revetments |
|  |  |  |  |  | 3,663,003.66 | | 5,250,000 | 660,000 | 1,440,000 |

**Supplementary Table S2 |** Costs of goods and services for the sustainable development of pasture space and transhumance corridors for livestock

| **Actions** | **Unit / threshold** | **Unit Cost (USD)** |
| --- | --- | --- |
| Identification of key stakeholders, including youth and women (e.g., landowners, local authorities, advocates) for a target grazing area of 250 ha | 50 people | 1,319.53 |
| Materialization with concrete signpost of access tracks to water points every 250 m alternated | 1 km | 879.68 |
| Securing a grazing area of 250 ha. | - | 2,932.29 |
| Identification and negotiation of transhumance corridors | 50 people | 3,665.36 |
| Materialization of transhumance corridors using a signpost at every 500 m | 1 km | 1,099.61 |
| Creation of multiple stakeholders for consultation / dialogue on transhumance corridors | 100 people | 7,330.73 |
| Building Legal Framework, Monitoring/Evaluation, and Management System of Rangeland/Pastoral Resources |  | - |
| Regeneration of Grazing Areas of Corridors | 1 ha | 1,466.14 |
| **TOTAL** | | **18,693.37** |

**References**

1. OECD/SWAC. Peuplement, marché et sécurité alimentaire, Cahiers de l'Afrique de l'Ouest, OECD Publishing, Paris, <https://doi.org/10.1787/9789264187412-fr> (2013).
2. Jakeman A.J., Littlewood I.G., & Whitehead P. G. Computation of the Instantaneous Unit Hydrograph and Identifiable Component Flows with Application to Two Small Upland Catchments, Journal of Hydrology, Volume117, Pages 275-300 (1990).
3. Jakeman A.J. & Hornberger G.M. How Much Complexity is Warranted in a Rainfall-Runoff Model?, Water Resource Research, Volume 29, 2637-2649 (1993).
4. Croke B.F.W. & Jakeman A.J. A Catchment Moisture Deficit module for the IHACRES rainfall-runoff model, Environmental Modelling and Software, volume 19, 1-5 (2004).
5. Oyerinde G. T., Wisser D., Hountondji F. C., Odofin A. J., Lawin A. E., Afouda A., & Diekkrüger B. Quantifying uncertainties in modeling climate change impacts on hydropower production. Climate, 4(3), 34 (2016).
6. Perrin C., Michel C. and Andreassian V. Improvement of a parsimonious model for streamflow simulation, Journal of Hydrology, Volume 279, Pages 275– 289, doi:10.1016/S0022‐1694(03)00225‐7 (2003).
7. Knoben W.J.M., Freer J.E., and Woods R. A. Technical note: Inherent benchmark or not? Comparing Nash–Sutcliffe and Kling-Gupta efficiency scores, Hydrological Earth System Science, Volume 23, Pages 4323-4331, <https://doi.org/10.5194/hess-23-4323-2019> (2019).
8. UEMOA, & IUCN. Regional shoreline monitoring study and a management scheme for the West African coastal Area, UEMOA, IUCN, 56p (2010).
9. Aman A., Tano R.A., Toualy E., Silué F., Appeaning Addo K., Folorunsho R. Physical forcing induced coastal vulnerability along the Gulf of Guinea. J. Environ Prot 10:1194–1211 (2019).
10. Thior M., Sané T., Dièye E-HB, Sy O., Cissokho D., Ba B.D., & Descroix L. Coastline dynamics of the northern lower Casamance (Senegal) and southern Gambia littoral from 1968 to 2017. J Afr Earth Sci 160:103611(2019).
11. Angnuureng D.B., Jayson-Quashigah P-N, Almar R., Stieglitz T.C., Anthony EJ, Aheto D.W., Appeaning Addo K. Application of shore-based video and uncrewed aerial vehicles (drones): complementary tools for beach studies. Remote Sens 12:394–413 (2020).
12. WACA. Effects of Climate Change on Coastal Erosion and Flooding in Benin, Côte d’Ivoire, Mauritania, Senegal, and Togo. Technical Report. West Africa Coastal Areas Program (WACA), 131p (2020).
